# The Amblyopia Produced by Certain Drugs

**Published:** 1908-05-02

**Authors:** 


### SPECIAL ARTICLE.

### THE AMBLYOPIA PRODUCED BY CERTAIN DRUGS.

### ALCOHOL.

Acute alcoholic amblyopia may be passed over in a few words; it consists in the temporary aberration of action of one or more of the external muscles of the eyeball, giving rise to the well-known double-vision of the intoxicated. This symptom soon passes off; and when the drunken man has slept away his stupor his eyes are practically normal again.

Lasting disorders of vision from acute alcoholic toxæmia are very uncommon from ordinary fluids containing ethyl-alcohol. The same does not hold good of methyl-alcohol, however, which is consumed in some parts in the form of various liqueurs. Buller and Wood have recorded 122 cases of sudden death, and 153 cases of bilateral and incurable blindness in America within one decade, all due to acute poisoning by methyl-alcohol.

Chronic alcoholism requires a rather longer discussion. The ocular changes are remarkably similar to those which, as we shall see, may be due to excessive use of tobacco. The following is a summary of their chief features:—(1) There is seldom anything wrong to be seen in the optic disc or in the retina. (2) Vision in the centre of the field is always affected upon both sides if at all, and the degree of affection is symmetrical. (3) The field of vision for white is normal, or nearly so. (4) In the centre of the visual field, or near the centre, there is at first a scotoma for colours, and later an absolute scotoma; that is to say, in a very small area of the visual field in or near the centre there is at first a diminished power to distinguish colours, and later there may be inability to see with that part of the retina at all.

Attempts have been made to distinguish between alcoholic and tobacco amblyopias clinically; for instance, it has been stated that alcohol more often leads to a pericentral scotoma immediately around the fixation point, whereas tobacco is asserted to cause a paracentral scotoma which stretches out in the form of an oval towards the temporal side. The distinction, however, cannot be maintained; each form of scotoma may be due to either poison.

It was not known until quite recently why these peculiar changes in the field of vision should come about. It has now been shown that there is a particular bundle in the optic nerve, which has been styled the papillo-macular bundle of Samelsohn, of fibres derived from the region between the optic papilla and the macula lutea. Although it is derived from only so small a part of the entire retina, this bundle occupies the whole of the temporal third of the papilla.

There exists, therefore, in the optic nerve a special bundle of fibres which convey impulses from the macula lutea; and when one finds blindness in this region only, all the rest of the retina being normal, one can assume with fair certainty that this bundle is affected. Why it should be picked out in this way is not so obvious. Some regard it as an atrophy from the start; others hold that the atrophy is preceded by an inflammation of the interstitial tissue analogous to a retro-bulbar neuritis. There are some who believe that the primary trouble is neither in nor around the nerve fibres themselves, but in the ganglionic cells of the retina. There are no means of deciding between these various views at present, in spite of experiments upon monkeys. The point is that either alcohol or tobacco can in some way exert a baneful influence upon the elements of this particular bundle of optic nerve fibres.

The degree to which sight is impaired is very variable. If the trouble has not already gone far when it is first detected there is a possibility of its resolving again, provided always that no fresh excesses occur. If the atrophy has already advanced far, and if teetotalism cannot be maintained, the impairment of sight will reach a point at which it will be impossible to do more than distinguish fingers at a yard or two's distance. It is very uncommon for the blindness to become absolute. Alcoholic amblyopia may arise from the misuse of any variety of spirituous liquors, and yet it is hardly ever met with before forty. It would seem, therefore, that it takes a good many years of alcoholism to start it. It generally sets in at the time when the patient begins to suffer in other ways as well, particularly from gastro-intestinal trouble. It is distinctly more common amongst lower-class patients, but not absolutely confined to them. It cannot be attributed entirely to raw spirits or to fusel oil and its allies, for there are cases on record in which it has appeared in men who have taken only sound wines or strong beers, though in excess.

### TOBACCO.

The phenomena of tobacco amblyopia are almost identical with those of alcoholic patients who have not smoked; therefore, as many patients indulge in both poisons to excess, there will often be doubts as to which of the two is to be blamed in a particular case.

A collection of 138 cases made by Uhthoff teaches us that in about 38 per cent. the cause is chiefly alcohol, that in about 33 per cent. the trouble is due to both alcohol and tobacco, that in 18 per cent. tobacco alone is at the root of the mischief; in the remaining 11 per cent. other poisonous substances, such as carbon bisulphide, have also played a part.

It must be granted, however, that the number of cases in which amblyopia occurs is remarkably small when one bears in mind how many people smoke too much, drink too much, or do both.

Tobacco amblyopia is very seldom the first symptom of poisoning by this substance; it nearly always comes on when other symptoms have already appeared. In the great majority of the cases there have been cardiac symptoms from affection of the nervous mechanism of the heart. The commonest of these are palpitation, sensations as though the heart stopped beating for short intervals every now and then, or gripping pains in the precordial area. The dimness of vision develops quite slowly, increases gradually to a certain point, and there remains stationary. Complete blindness never results.

The commonest mode of poisoning is by smoking. In a few instances the trouble has followed the chewing of tobacco; it has never resulted from taking tobacco in the form of snuff. It may, however, result from the inhalation of tobacco dust in cigarette or cigar factories.

The best known poison in tobacco is nicotine, but it is by no means the only one present. Different varieties of tobacco contain very different proportions of nicotine, as the following figures show:—

| | Per cent. of Nicotine. |
|---|---|
| Fine Havannah tobacco contains ... ... | 2 |
| Alsatian " " ... ... | 3·3 |
| Turkish " " ... | only 1 to 1·25 |
| Virginian " " ... | as much as 6·9 |

Cigars and cigarettes are far more commonly the cause of the amblyopia than are pipes. The kind of tobacco smoked, the way it is smoked, and the time at and in which it is smoked, are all more important than are the actual quantities consumed. It is almost impossible to say precisely how much tobacco can be smoked with impunity; individuals vary enormously in this respect. But the severe forms of amblyopia are very seldom seen in persons who smoke less than an ounce a day, or five or six ordinary cigars.

The prognosis of tobacco amblyopia can generally be regarded as favourable, provided that the patient will forego all smoking. Here, as in the case of the morphia habit, there are two methods of giving it up—the sudden and the gradual. The latter is upon the whole the easiest and best. If a habitual smoker suddenly gives up the habit, he becomes, so to speak, thrown upon his beam ends; his plight is such that he is extremely apt to take to his smoking again. There are many such persons who can at once reduce their daily quantity to half, and then in a fortnight or so to a quarter. If the sight already begins to improve under this *régime*, it would really be too hard to insist on absolute abstention.

It is a great help in many cases for the patient to have either small jujubes, or else some good chewing-gum, to put into his mouth when the craving for a smoke becomes very strong.

It is quite certain that much may be done to diminish the risk of tobacco amblyopia by paying attention to certain points of personal hygiene. For instance, a rule should be made never to smoke upon an empty stomach, but as far as possible only after meals. It is absolutely bad to smoke before dinner, and equally bad to smoke late at night to keep awake at one's work. It should also be forbidden to chew the cigar between the teeth as many smokers are wont to do. The tobacco-leaf becomes sodden, and harmful substances dissolve in the saliva. If that saliva is swallowed, these substances become absorbed from the stomach. Spitting during smoking, nasty habit though it is, is in a sense a safeguard to the smoker.

It is similarly most unwise to go on smoking a cigar right up to its moistened end, both for the above reason and because there arises from that habit the local irritation of the tongue and lips, and the chronic pharyngitis from which so many excessive smokers suffer. It is a pity that the holder is not in more general use for cigars, especially when the latter have been smoked over half-way through.

### QUININE.

Shortly after quinine first came to be used in quantity, severe disturbances of vision arose from it. These were at first attributed to the malaria for which the quinine was being taken. It was soon shown, however, that those who had never had malaria, but who had taken quinine in large quantities, developed amblyopia. The case recorded by Giacomini in 1841 is classical. This observer had a patient who took 2¼ drachms of quinine sulphate at a single dose. He became comatose, and when he recovered consciousness he was both deaf and blind; the latter afflictions lasted a long time, though sight was ultimately recovered.

Experiments have been made with quinine in dogs, and it has been found that one can constantly produce both deafness and blindness for the time being, though recovery from both is usual.

The immediate cause of this blindness remained quite obscure for a long time, because the cases almost all occurred abroad, and the ophthalmoscope was not often used. It was noted that the pupils were widely dilated and fixed, that the eyes exhibited no light reflex, and that the optic papillæ looked atrophic. This, however, was chiefly in cases of some standing. In recent cases the ophthalmoscope shows quite a different picture—namely, that of *ischœmia retinæ*. The papilla is obscure, its edge no longer quite clear, and all the retinal vessels, both arteries and veins, are in the highest degree attenuated; indeed, the appearances are such as are commonly associated with embolism of the arteria centralis retinæ. The reason of this is not a thrombosis but a spasmodic contraction of all the vessels, which presently passes off again in favourable cases, and ends in complete recovery. In a few very unfavourable cases complete and permanent obliteration of the vessels has been recorded.

Quinine amblyopia is, therefore, of an entirely different pathology to that of tobacco or of alcohol. Whereas the latter produce degeneration in the papillo-macular bundle of optic nerve fibres, quinine leads to a powerful and spasmodic contraction of all the blood-vessels in the retina, and it depends upon the duration and severity of this vaso-constriction, whether recovery can be complete or only partial.

Quinine amaurosis is nowadays a very rare condition, fortunately. It has been recorded in a patient who took one drachm of quinine sulphate in twenty-four hours, but many persons can take such a dose without the eyes suffering. As regards treatment, the main thing is to desist at once from taking quinine. Amyl nitrite may be inhaled, with a view to bringing about vaso-dilatation; and subcutaneous injections of strychnine have been advocated.

### FILIX MAS.

It has long been known that male fern occasionally causes disorder of vision, and in rare cases the trouble has been permanent. There was an epidemic of ankylostomiasis in Rhenish Westphalia not long since, during which no fewer than 22,000 persons came under treatment. Some of the patients were excessively anæmic, whilst others, though they had the worms in their intestines, were constitutionally well. They received five grains of calomel at night, instead of castor oil, which seems to be harmful in these cases, and next day 2½ drachms of the extract of filix mas. Four of the 22,000 persons became suddenly and lastingly blind.

At first the patients so affected were comatose, with widely dilated and fixed pupils, and the ophthalmoscopic appearances of the retinæ were almost identical with that of sudden embolism of the central artery. The fundus exhibited intense white œdema of the retina right out to its periphery, and in the œdema the vessels were here and there lost to sight; the optic disc and the macula lutea were indistinguishable; the arteries were narrowed to the finest threads, whilst the veins were wide and tortuous. In such parts of the vessels as could be continuously traced the blood column seemed to be interrupted here and there. The patients did not recover their sight, and in the course of time the appearances of optic nerve atrophy developed.

It would seem, therefore, that filix mas can sometimes act like quinine does, in producing a spasmodic contraction of the retinal vessels and thus rendering the retina functionless. Hitherto it has generally been assumed that the drug acts either upon the cortical centre for vision, or else upon the optic nerve itself, leading to atrophy. It is now clear that the atrophy is a secondary phenomenon, and that the primary effect is one of extreme vaso-constriction of the retinal vessels.

